# Implementation of the GSA KAER Toolkit in a Large Clinic System: Workflow Modifications and EMR Tools

**DOI:** 10.1093/geroni/igab046.1337

**Published:** 2021-12-17

**Authors:** Jaqueline Raetz, Barak Gaster, Basia Belza, Monica Zigman Suchsland, Judit Illes, Benjamin Olivari, Lisa McGuire, Annette Fitzpatrick

**Affiliations:** 1 University of Washington, Seattle, Washington, United States; 2 University of Washington, Seattle, University of Washington, Washington, United States; 3 The Gerontological Society of America, Washington, District of Columbia, United States; 4 Centers for Disease Control and Prevention (CDC), Atlanta, Georgia, United States; 5 CDC, Atlanta, Georgia, United States

## Abstract

We implemented the KAER toolkit in the University of Washington primary care clinics. In this session we share the workflows implemented to promote the KAER model and share the tools we developed within EPIC, the system's electronic medical record (EMR). We collaborated with clinic staff to develop interdisciplinary workflows including: training patient service representatives, social workers, nurses, and medical assistants (MAs) about 'red flags;' training medical assistants to complete the Patient Health Questionnaire (PHQ-9) and Montreal Cognitive Assessment (MoCA); and assuring they are appropriately entered into flowsheets in EPIC. We created a checklist (EPIC 'SmartPhrase') and educated the clinics' interdisciplinary teams to utilize it within their scope of practice. Additionally, we created an order set (EPIC 'SmartSet') of commonly ordered tests and referrals to expedite evaluation of patients with suspected cognitive impairment. Lastly, we created a direct link from our EMR to our website containing community resources.

